# Response to Bates

**DOI:** 10.1093/jncics/pkag064

**Published:** 2026-06-19

**Authors:** Gabriel A Benavidez, Ami E Sedani, Tisha M Felder, Matthew Asare, Charles R Rogers

**Affiliations:** Department of Public Health, Robbins College of Health and Human Sciences, Baylor University, Waco, TX, United States; Department of Epidemiology, UTHealth Houston School of Public Health, Dallas, TX, United States; Department of Biobehavioral Health & Nursing Science, Arnold School of Public Health, University of South Carolina, Columbia, SC, United States; Department of Public Health, Robbins College of Health and Human Sciences, Baylor University, Waco, TX, United States; Men’s Health Inequities Research Lab, Atlanta, GA, United States

We thank Mr Bates for their assessment of our study,^1^ and the opportunity to clarify considerations related to changes in Behavioral Risk Factor Surveillance System (BRFSS) cervical cancer screening ascertainment. As noted in our original discussion, interpretation of recent cervical cancer screening estimates requires caution given reliance on self-report and evolving survey design, considerations that include, but are not limited to, the 2022 modification to question structure.

We agree that the revised 2-stage cervical cancer screening item introduced in 2022 may influence respondent interpretation by requiring recognition of screening modalities (Papanicolaou [Pap] or human papillomavirus [HPV] testing). Prior evidence of gaps in HPV-related knowledge supports the possibility of measurement error.^2^ However, the extent to which differences in respondent knowledge translate into systematic underreporting in BRFSS, and whether such effects changed meaningfully over time, remains uncertain.

We therefore caution against interpreting the 2022 decline in cervical cancer screening prevalence primarily as a measurement artifact. From a temporal perspective, the observed rebound in screening estimates between 2022 and 2024 despite continued use of the revised question structure is not fully consistent with a persistent survey effect. More broadly, declines in screening were evident across multiple cancers and were observed in prior data sources predating both the COVID-19 pandemic and recent BRFSS changes, suggesting that broader secular and pandemic-related factors remain important contributors.^3^

Importantly, our primary objective was to examine rural–urban differences and temporal patterns in being up-to-date with screening across multiple cancers, rather than to estimate cervical cancer screening prevalence in isolation. In this context, the consistency of directional trends including declines observed for breast and colorectal cancer screening, where question structure did not change, and the narrowing of rural–urban differences remain informative, even in the presence of measurement limitations affecting any single outcome.

Finally, although self-reported data have recognized limitations, alternative data sources such as electronic health records and claims data also incompletely capture screening delivered across fragmented care settings,^4^ particularly among younger and more mobile populations. As such, all available data sources for screening surveillance reflect tradeoffs between measurement error and population coverage. Although these considerations highlight limitations, this should not preclude the use of BRFSS for population-level surveillance, particularly when trends are interpreted in the context of known measurement constraints. These considerations further underscore the importance of continued methodological evaluation and triangulation across complementary surveillance systems when interpreting population-level screening trends. We agree that caution is warranted in interpreting the magnitude of recent cervical cancer screening estimates; however, the totality of evidence supports our conclusion that screening uptake remains below pre-pandemic levels, underscoring persistent challenges in achieving guideline-concordant cancer screening.

## Data Availability

There are no new data associated with this article.
